# Development and validation of a risk prediction model for autologous arteriovenous fistula thrombosis in patients receiving maintenance hemodialysis

**DOI:** 10.1080/0886022X.2025.2477832

**Published:** 2025-05-13

**Authors:** Jinping Ying, Genlian Cai, Yujiao Zhang, Minmin Zhu, Mengyan Pan, Ping Zhang

**Affiliations:** ^a^ Department of Nursing, The First Affiliated Hospital, Zhejiang University School of Medicine, Hangzhou, China; ^b^ Kidney Disease Center, The First Affiliated Hospital, Zhejiang University School of Medicine, Hangzhou, China

**Keywords:** Hemodialysis, native arteriovenous fistula, thrombosis, nomogram

## Abstract

**Background:**

Thrombosis can lead to fistula failure and affect the smooth progress of hemodialysis. This study aims to develop and validate a nomogram for predicting the risk of autologous arteriovenous fistula thrombosis in patients undergoing maintenance hemodialysis.

**Methods:**

A total of 1,016 patients who underwent hemodialysis at a tertiary A hospital in East China from February 2020 to March 2024 were retrospectively enrolled. The participants were randomly divided into a training set (711 people) and a validation set (305 people) at a ratio of 7:3. A risk prediction model was established according to the results of multivariate logistic regression analysis. The performance of the model was evaluated with the area under the receiver operating characteristic (ROC) curve (AUC), calibration curve analysis, the Hosmer–Lemeshow (H-L) test and decision curve analysis (DCA).

**Results:**

The incidence of autologous arteriovenous fistula thrombosis in patients on maintenance hemodialysis was 32%. High-sensitivity C-reactive protein (hs-CRP), catheterization history, hemodialysis duration, autologous arteriovenous fistula stenosis and non-high-density lipoprotein cholesterol (non-HDL-C) were independent risk factors for autologous arteriovenous fistula thrombosis. These five predictors were used to construct a predictive nomogram. The AUC was 0.818 in the training set and 0.826 in the validation set. The calibration curve of the nomogram was close to the standard curve, indicating that the model was well calibrated. The DCA results confirmed that the model provided good net clinical benefits.

**Conclusion:**

In this study, a predictive nomogram for arteriovenous fistula thrombosis was established and validated.

## HOW DOES THIS ARTICLE CONTRIBUTE TO THE WIDER GLOBAL COMMUNITY?

This study established a nomogram to predict autogenous arteriovenous fistula thrombosis. The model has high predictive ability and can aid in identifying individuals at high risk of developing autologous arteriovenous fistula thrombosis. The model can also assist medical personnel in designing and implementing appropriate early education and intervention programs to prevent this condition.

## 1. Introduction

Renal replacement therapy (RRT) is a life-sustaining treatment for patients with end-stage renal disease (ESRD). Maintenance hemodialysis (MHD) is the most common method of RRT, accounting for approximately 89% of procedures [1]. The prerequisite for receiving MHD is long-term, stable and reliable vascular access because the quality of vascular access directly affects the quality of patients’ dialysis [2]. Different types of vascular access can be established, including autogenous arteriovenous fistulas (AVFs), arteriovenous grafts (AVGs) and central venous catheters (CVCs) [3]. An autologous AVF refers to a method of vascular access in which adjacent autologous arteries and veins are surgically anastomosed to allow hemodialysis. Compared with AVGs and CVCs, AVFs are more convenient and safer, allow more stable blood flow, have a longer service life and are associated with fewer complications. Thus making AVFs currently the preferred method of vascular access for hemodialysis treatment [4]. However, AVFs are also associated with several drawbacks. Previous studies have shown AVF patency rates of 71.2–93.9% at one year [5,6], 64.2–92.7% at two years [5,7], and 4.1–43% after five years [6,7]. This decrease in AVF patency has been attributed primarily to AVF thrombosis [8]. This condition can hinder the hemodialysis process, leading to complications such as hyperkalemia and heart failure and increasing the use of central venous catheters, the rate of hospitalization, and mortality in dialysis patients [1]. Therefore, preventing AVF thrombosis is crucial for ensuring successful hemodialysis.

Previous studies have investigated the risk factors associated with AVF thrombosis. The causes can be grouped into sociodemographic factors (older age [9,10], female sex [9,11], a low education level [12], smoking, poor treatment compliance, and a lack of self-care ability [11]), disease-related factors (hypotension [11], diabetes [11], coronary artery disease [13], hypertension [9], and peripheral vascular disease [10]), laboratory indices (high hemoglobin level [9], platelet count, lymphocyte count and platelet–lymphocyte ratio [14], low-density lipoprotein cholesterol (LDL-C) level, high-sensitivity C-reactive protein (hs-CRP) level [15], fibrinogen level [6], blood calcium and phosphorus balance disorders, and increased parathyroid hormone level [16]), and fistula-related factors (AVF duration, the degree of AVF stenosis [17] and previous placement of ipsilateral or bilateral central venous catheterization (CVC) [13]). Given the low AVF patency rates described above, risk factors for AVF thrombosis must be identified to prolong the service life of the AVF, ensure early intervention for a smooth, high-quality hemodialysis process, and minimize the amount of pain experienced by patients.

To date, most studies of AVF thrombosis in MHD patients have focused on the analysis of influencing factors. However,the sensitivity and accuracy of these factors for AVF thrombosis identification are not satisfactory and few studies have investigated risk prediction models for AVF thrombosis in MHD patients. Therefore, in this study, we developed a preliminary nomogram prediction model for AVF thrombosis in MHD patients with the aim of providing strong evidence-based support for medical and nursing staff to identify populations of MHD patients at high risk for AVF thrombosis and conduct targeted prevention and intervention.

## 2. Method

### 2.1. Study design and participants

This study was conducted following the Transparent Reporting of a Multivariable Prediction Model for Individual Prognosis or Diagnosis (TRIPOD) statement. This was a retrospective study that included 1,016 patients who received hemodialysis at a tertiary-level A hospital in East China from February 2020 to March 2024. The inclusion criteria were age ≥18 years and regular hemodialysis treatment for ≥3 months. The exclusion criteria were CVC- or AVG-based vascular access, an AVF created less than 3 months prior, and a history of AVF thrombosis before the start of the study. A total of 27 potential predictive risk factors for AVF thrombosis were included in this study. In accordance with the procedures described by Riley et al. [18], the sample size for the risk prediction model should be at least 10 times the number of independent variables. Previous studies [19] have shown that the incidence of AVF thrombosis in hemodialysis patients is 38.89%. Assuming that 10% of the samples would be invalid, the minimum sample size required for this study was 27 × 10 ÷ 38.89%÷(1–10%)=771 patients. A total of 1,016 patients were ultimately included in this study and divided into training and verification sets at a 7:3 ratio.

### 2.2. Data collection

The data for this study were derived from the electronic medical records system, laboratory system, and hemodialysis system of the hospital. Four members of the research team collected sociodemographic data, disease-related data, laboratory test indicators, and vascular access-related data. Microsoft Excel was used to establish a database for double data entry and double verification.Among the 27 predictor variables, only parathyroid hormone, KT/V, hs-CRP, and D-dimer had missing data (less than 5% for each). Before model construction, the missing data for continuous variables were added 10 times by multiple interpolation [20], and the set of data with the smallest AIC and BIC values was selected for subsequent analysis.

#### 2.2.1. Diagnostic criteria for autologous AVF thrombosis

The diagnostic criteria for autologous AVF thrombosis [21,22] include the following: (1) absence of the original fistular tremor or pulsation, (2) absence of a fistular murmur and hardening of the associated vein preventing compression, (3) possible local inflammation, swelling and pain, and (4) color Doppler ultrasound that reveals low-density or medium-density echoes and no blood flow signal in the AVF vessel lumen.

#### 2.2.2. Candidate predictors

On the basis of previous studies [9–13,15–17], our experience in clinical practice and expert consultation, the research team discussed and ultimately included 27 predictive factors divided into four groups: sociodemographic factors (age, sex, smoking history, BMI, education level, and marital status); disease-related factors (hypertension, hypotension, history of diabetes, history of cardiovascular disease, history of tumors, and duration of hemodialysis); laboratory test indicators (platelet–lymphocyte ratio, serum albumin level, hemoglobin level, blood calcium level, blood phosphorus level, parathyroid hormone level, non-HDL-C level, hs-CRP level, urea clearance index, fibrinogen level, D-dimer level, and random blood glucose); and vascular access-related factors (history of CVC, AVF use time, and AVF stenosis). For the nonthrombosis group, these data were collected as close to the end of the study period as possible, whereas for patients with AVF thrombosis, the data were collected from the latest date(s) prior to the day thrombosis was first identified.

Non-HDL-C is a cholesterol that is not affected by factors such as diet and is calculated by subtracting the HDL-C level from the total cholesterol level [23].

According to the Kidney Disease Outcome Quality Initiative (K/DOQI) guidelines [24], intradialytic hypotension (IDH) was defined as a decrease in systolic blood pressure by 20 mmHg or a decrease in mean arterial pressure by 10 mmHg during dialysis compared with predialysis levels accompanied by clinical symptoms such as yawning, nausea, vomiting, and muscle cramps and the need for intervention measures such as injection of high-concentration glucose, placement in a head-down position, or even temporary suspension of hemodialysis.

### 2.3. Statistical analysis

Statistical analysis was performed with R version 4.2.1 (R Foundation for Statistical Computing, Vienna, Austria). The entire set was randomly divided into a training set and a validation set at a ratio of 7:3 [25]. Multivariate logistic regression analysis was used to screen predictors and construct a model with the training set variables, and the validation set was used to verify the ability of the constructed model to predict the risk of AVF thrombosis.

Continuous variables with a normal distribution were expressed as the mean ± standard deviation (χ ± s), and the independent-sample t test was used for between-group comparisons. Variables with a nonnormal distribution were expressed as medians and interquartile ranges [M (P25, P75)], and the Mann–Whitney U test was used for between-group comparisons. Counting data were expressed as numbers (%), and the chi-square test or Fisher’s exact probability test was used for between-group comparisons. The Wilcoxon rank-sum test was used to compare rank data between the groups.

Training set variables with *p* < 0.05 in the univariate analysis were included in the multivariate logistic regression analysis. The backward stepwise regression method was used to determine the independent risk factors for AVF thrombosis and to construct a prediction model, which was subsequently visualized in the form of a nomogram. The area under the receiver operating characteristic (ROC) curve (AUC), calibration curve analysis, Hosmer–Lemeshow (H-L) test and decision curve analysis (DCA) were used to evaluate the performance of the model. *p* < 0.05 indicated statistical significance.

### 2.4. Ethical considerations

This study was approved by the Ethics Committee (review round 2022, research no. 136-Fast). Due to the retrospective nature of the study, the need for informed consent from patients was waived.

## 3. Results

### 3.1. Characteristics of the participants

A total of 1,194 MHD patients were initially recruited for this study. Of these patients, 76 patients with central venous catheterization, 43 patients with graft arteriovenous fistulas, 24 patients who had AVFs for <3 months, 15 patients on dialysis for <3 months, 9 patients with a history of AVF thrombosis before the start of the study, and 11 patients with missing values in more than 20% of their medical records were excluded. Finally, 1016 MHD patients were included and divided into a training set (711 patients) and a validation set (305 patients) at a ratio of 7:3. Among the 1016 patients, 664 (65%) were male, 352 (35%) were female, and the average age was 59 (48, 69) years. A total of 324 patients (32%) were diagnosed with thrombosis, whereas the other 692 patients (68%) were not. The baseline data of the two groups of patients are shown in Table 1.

**Table 1.** Baseline characteristics of the patients in the training and validation sets.

Characteristics	Total (*n* = 1,016)	Validation set (*n* = 305)	Training set (*n* = 711)	*P* value
**Socio-demographic**				
Age (years)	59 (48, 69)	60 (49, 70)	59 (48, 69)	0.523
Male n (%)	664 (65)	188 (62)	476 (67)	0.119
Smoking history n (%)	185 (18)	69 (23)	116 (16)	0.021
BMI (kg/m^2^)	21.56 (19.47, 24.31)	21.28 (19.44, 23.8)	21.73 (19.53, 24.43)	0.126
Education, n (%)				0.961
Primary	259 (25)	76 (25)	183 (26)	
Secondary	301 (30)	91 (30)	210 (30)	
High school	229 (23)	67 (22)	162 (23)	
College or above	227 (22)	71 (23)	156 (22)	
Married n (%)	929 (91)	278 (91)	651 (92)	0.925
**Disease-related**				
Primary disease n (%)				0.351
Glomerulonephritis	719 (71)	207 (68)	512 (72)	
Diabetic nephropathy	166 (16)	57 (19)	109 (15)	
Other	131 (13)	41 (13)	90 (13)	
Diabetes mellitus n (%)	323 (32)	104 (34)	219 (31)	0.337
Hypotension n (%)	226 (22)	64 (21)	162 (23)	0.582
Hypertension n (%)	703 (69)	223 (73)	480 (68)	0.089
History of tumors n (%)	76 (7)	21 (7)	55 (8)	0.732
History of CVD n (%)	113 (11)	35 (11)	78 (11)	0.9
Hemodialysis duration (months)	37 (20, 90.25)	42 (22, 95)	35 (18, 88.5)	0.083
**Laboratory**				
hs-CRP (mg/L)	2.29 (0.85, 5.61)	2.3 (0.8, 5.95)	2.28 (0.87, 5.46)	0.988
Non-HDL-C (mmol/L)	2.24 (1.79, 2.94)	2.25 (1.76, 2.98)	2.24 (1.8, 2.91)	0.642
Hemoglobin (g/L)	114 (104, 122)	113 (105, 122)	114 (104, 122)	0.667
PLR	165.5 (126.82, 215.69)	164.34 (125.98, 215.12)	165.62 (127.64, 216.53)	0.746
Serum phosphorus (mmol/L)	1.76 (1.43, 2.14)	1.78 (1.42, 2.16)	1.76 (1.43, 2.12)	0.643
Serum calcium (mmol/L)	2.24 (2.13, 2.36)	2.22 (2.13, 2.36)	2.25 (2.14, 2.37)	0.132
Serum albumin (g/L)	39.6 (37.4, 41.6)	39.3 (37, 41.6)	39.7 (37.6, 41.6)	0.102
Fibrinogen (g/L)	3.18 (2.68, 3.84)	3.17 (2.61, 3.86)	3.18 (2.7, 3.84)	0.542
PTH (pg/ml)	201.5 (113, 303)	206 (106, 302.5)	201 (114, 303)	0.797
Kt/V	1.49 (1.32, 1.7)	1.51 (1.32, 1.74)	1.48 (1.32, 1.68)	0.173
D-Dimer (µg/L FEU) n (%)				0.161
≤700	924 (91)	271 (89)	653 (92)	
>700	92 (9)	34 (11)	58 (8)	
**Vascular access-related**				
Stenotic AVF n (%)	414 (41)	128 (42)	286 (40)	0.654
History of CVC, n (%)	748 (74)	215 (70)	533 (75)	0.16
Duration of AVF use (months)	34 (16, 80.25)	38 (19, 89)	33 (15, 72.5)	0.044

Note: Non-HDL-C = Non-high-density lipoprotein cholesterol, hs-CRP = high-sensitivity C-reactive protein, PLR = platelet–lymphocyte ratio, CVD = cardiovascular disease, Kt/V = urea clearance index, AVF = arteriovenous fistula, PTH = parathyroid hormone, CVC = central venous catheterization.

### 3.2. Correlation analysis

Patients were divided into an AVF thrombosis group and a non-AVF thrombosis group according to whether they had AVF thrombosis. In the training set, the results of univariate analysis revealed that the serum albumin level, hs-CRP level, intubation history, hemodialysis duration, AVF stenosis, and non-HDL-C level were significantly different between the two groups (*p* < 0.05). Multicollinearity analysis was performed on the factors identified above. The results revealed that the variance inflation factors of all the variables were <2.0, indicating that there was no multicollinearity among them. The above variables were therefore included in the multivariable logistic regression analysis. The results revealed that hs-CRP [odds ratio (OR)=1.05)], history of CVC (OR = 1.924), hemodialysis duration (OR = 1.006), AVF stenosis (OR = 6.974), and non-HDL-C (OR = 1.835) were independent risk factors for AVF thrombosis (Tables 2 and 3).

**Table 2.** Univariate analyses of risk factors for arteriovenous fistula thrombosis in hemodialysis patients in the training set.

Characteristics	OR	CI	P
Age (years)	1.004	1.004 (0.993–1.015)	0.524
Sex	1.173	1.173 (0.837–1.653)	0.358
Smoking history	0.877	0.877 (0.562–1.345)	0.555
BMI (kg/m^2^)	0.961	0.961 (0.92–1.003)	0.071
Education			
Primary	Reference		
Secondary	0.912	0.912 (0.599–1.389)	0.668
High school	0.85	0.85 (0.54–1.335)	0.482
College or above	0.748	0.748 (0.469–1.187)	0.22
Marital status	0.842	0.842 (0.458–1.486)	0.565
Diabetes mellitus	1.085	1.085 (0.77–1.522)	0.638
Hypertension	0.974	0.974 (0.697–1.367)	0.877
Hypotension	0.729	0.729 (0.489–1.071)	0.113
History of tumors	1.153	1.153 (0.634–2.035)	0.63
History of CVD	1.32	1.32 (0.801–2.139)	0.267
Hemodialysis duration (months)	1.006	1.006 (1.003–1.008)	<0.001
hs-CRP (mg/L)	1.061	1.061 (1.041–1.085)	<0.001
Non-HDL-C (mmol/L)	1.69	1.69 (1.386–2.072)	<0.001
Hemoglobin (g/L)	0.992	0.992 (0.982–1.002)	0.126
PLR	1	1 (0.998–1.002)	0.837
Serum phosphorus (mmol/L)	1.097	1.097 (0.817–1.468)	0.537
Serum calcium (mmol/L)	0.607	0.607 (0.261–1.402)	0.243
Serum albumin (g/L)	0.931	0.931 (0.887–0.976)	0.003
Fibrinogen (g/L)	1.011	1.011 (0.852–1.196)	0.899
PTH (pg/ml)	1.001	1.001(1–1.001)	0.271
Kt/V	1.073	1.073 (0.611–1.877)	0.806
D-Dimer (µg/L FEU)	1.467	1.467 (0.836–2.532)	0.173
Stenotic AVF	7.033	7.033 (4.967–10.05)	<0.001
Duration AVF use	1	1 (0.999–1.001)	0.888
History of CVC	2.167	2.167 (1.457–3.292)	<0.001

Note: Non-HDL-C = Non-high-density lipoprotein cholesterol, hs-CRP = high-sensitivity C-reactive protein, PLR = platelet/lymphocyte ratio, CVD = cardiovascular disease, Kt/V = urea clearance index, AVF = arteriovenous fistula, PTH = parathyroid hormone, CVC = central venous catheterization.

**Table 3.** Multivariate analyses of risk factors for arteriovenous fistula thrombosis in hemodialysis patients in the training set.

Characteristics	*OR*	*95%CI*	*P*
(Intercept)	0.016	0.015 (0.001–0.178)	0.001
hs-CRP (mg/L)	1.05	1.049 (1.027–1.074)	<0.001
History of CVC	1.924	1.923 (1.205–3.132)	0.007
Hemodialysis duration (months)	1.006	1.006 (1.003–1.009)	<0.001
Stenotic AVF	6.974	6.973 (4.767–10.33)	<0.001
Non-HDL-C (mmol/L)	1.835	1.835 (1.454–2.337)	<0.001
Serum albumin (g/L)	0.994	0.993 (0.936–1.053)	0.83

Note: hs-CRP = high-sensitivity C-reactive protein, AVF = arteriovenous fistula, non-HDL-C = non-high-density lipoprotein cholesterol, CVC = central venous catheterization.

### 3.3. Nomogram for predicting autologous arteriovenous fistula thrombosis

**Figure 1.**
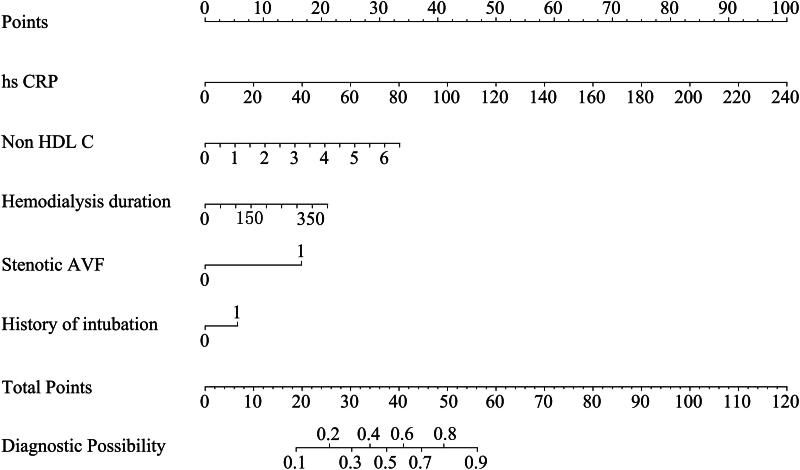
Nomogram for predicting AVF thrombosis in MHD patients. non-HDL-C = non-high-density lipoprotein cholesterol, hs-CRP = high-sensitivity C-reactive protein, AVF = arteriovenous fistula, CVC=central venous catheterization.

The risk prediction model for autogenous arteriovenous fistula thrombosis in MHD patients was established according to the prediction model formula: *p* = 1/(1+ e-y), where e is the base of the natural logarithm, Y=–4.153 + 0.048 × C-reactive protein +0.654 × catheterization history + 0.006 × hemodialysis duration +1.942 × stenosis history + 0.607 × non-high-density lipoprotein, and a visual histogram was drawn (Figure 1). Each risk factor was scored according to the score scale in the first row. The scores of all existing risk factors were then summed to obtain the total score, which was subsequently projected to the location on the ‘probability of AVF thrombosis’ line to identify the predicted probability. For this nomogram, the best probability cutoff value for diagnosing AVF thrombosis was 0.368. For example, in a female patient, the hs-CRP level was 20.3 mmol/L (8.1 points), the non-HDL-C level was 2.25 mmol/L (11.5 points), the patient had AVF stenosis (17 points), the patient had a history of CVC (6 points), and the duration of hemodialysis was 109 months (5.2 points). The total score for this patient was 8.1 + 11.5 + 17 + 6 + 5.3 = 47.8 points. Therefore, the probability that this patient would have thrombosis was approximately 77%. Because this value was greater than the optimal cutoff value of the model (0.368), this patient would be classified as being at high risk of developing AVF thrombosis and should receive appropriate interventions as early as possible.

### 3.4. Discrimination and calibration

**Figure 2.**
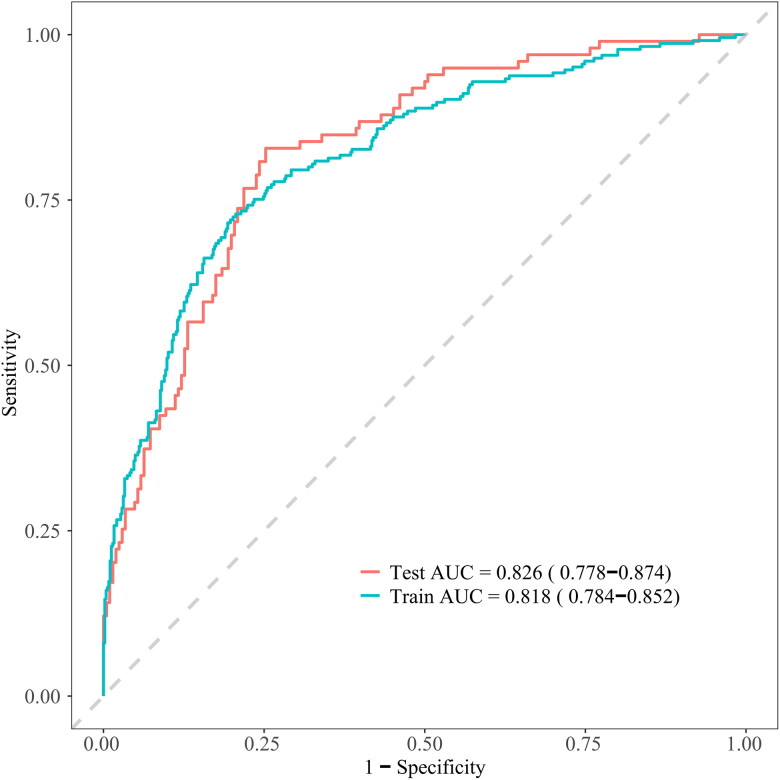
Receiver operating characteristic curves of the nomogram in the two sets.

**Figure 3.**
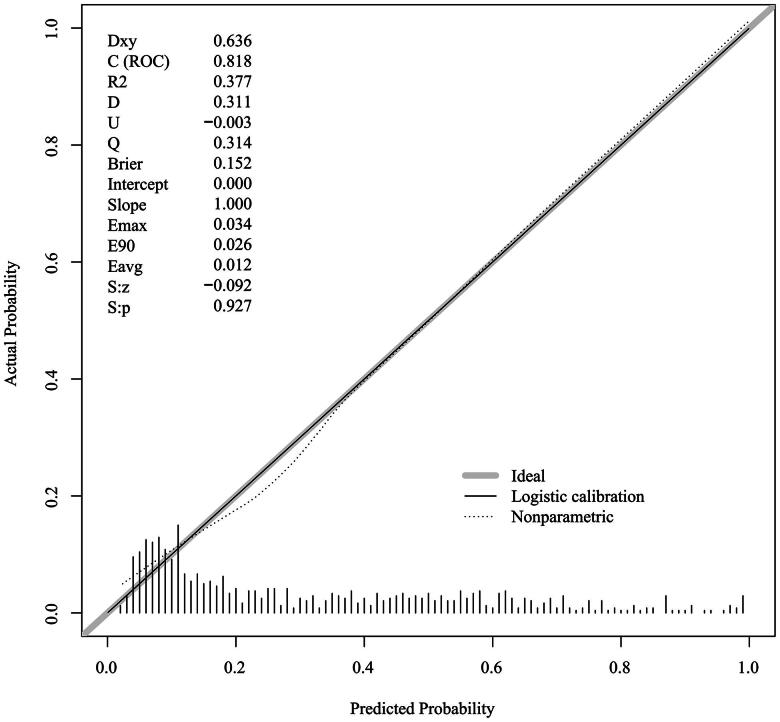
Calibration curve of the nomogram in the training set.

**Figure 4.**
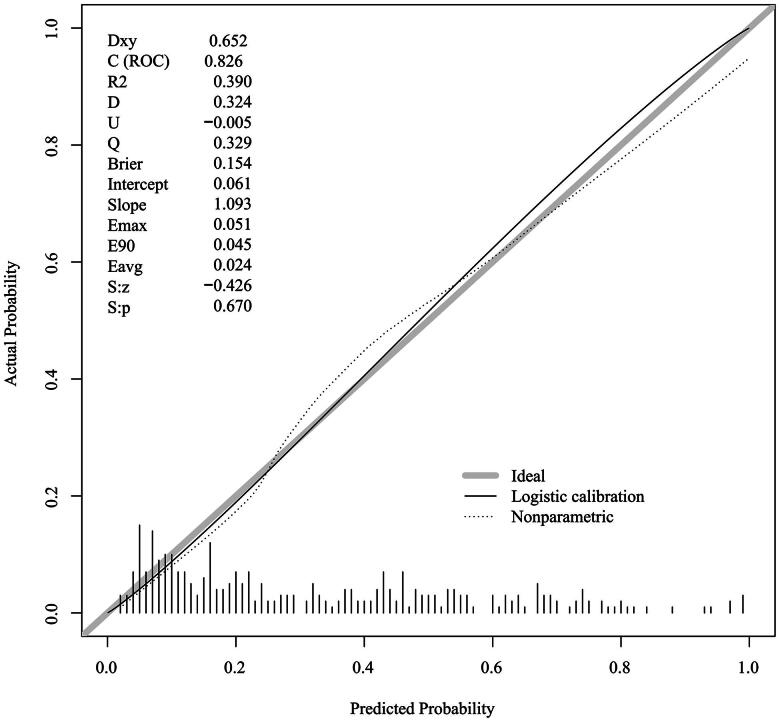
Calibration curve of the nomogram in the verification set.

After the predictive model was developed *via* the development set (*n*= 711), the validation set (*n*= 305) was employed to assess the model’s predictive performance. In the training set, the AUC was 0.818, the specificity was 79.4%, and the sensitivity was 72.9% (Figure 2). The calibration curve of the nomogram was close to the standard curve (Figure 3). The P value of the H–L test was 0.258, and the C index was 0.820 after 1000 rounds of repeated sampling with the bootstrap method. In the validation set, the AUC was 0.826, the specificity was 74.8%, and the sensitivity was 82.8% (Figure 2). The calibration curve of the nomogram was close to the standard curve (Figure 4). The P value of the H–L test was 0.632, and the C-index was 0.830. The accuracy rate after 1000 bootstrap samples for both groups was 77.4%. The results indicated that the model had good discriminative and calibration ability and could be used to predict the risk of AVF thrombosis in MHD patients.

### 3.5. Clinical practicality

**Figure 5.**
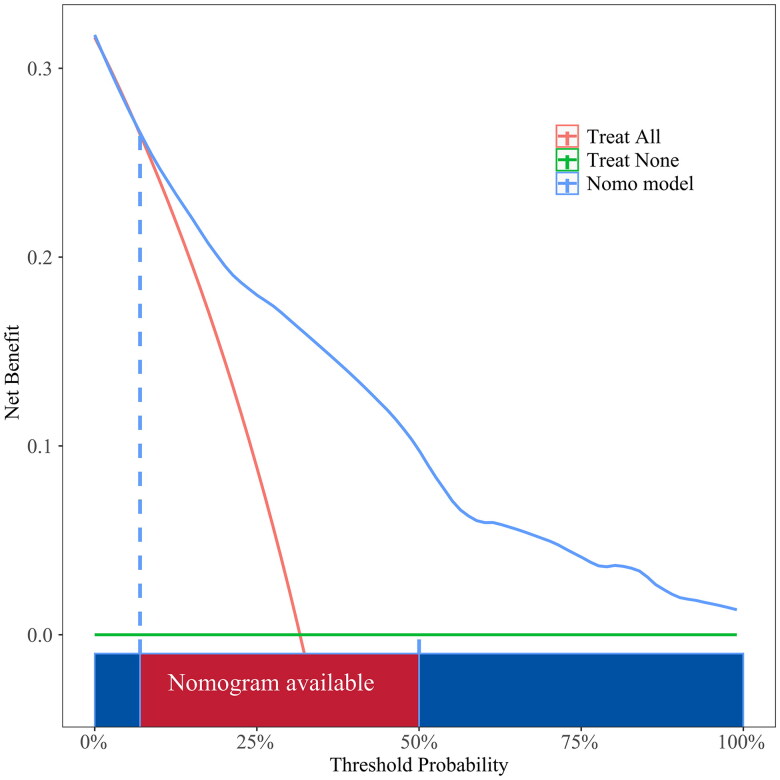
Decision curve analysis plot of the nomogram in the training set.

**Figure 6.**
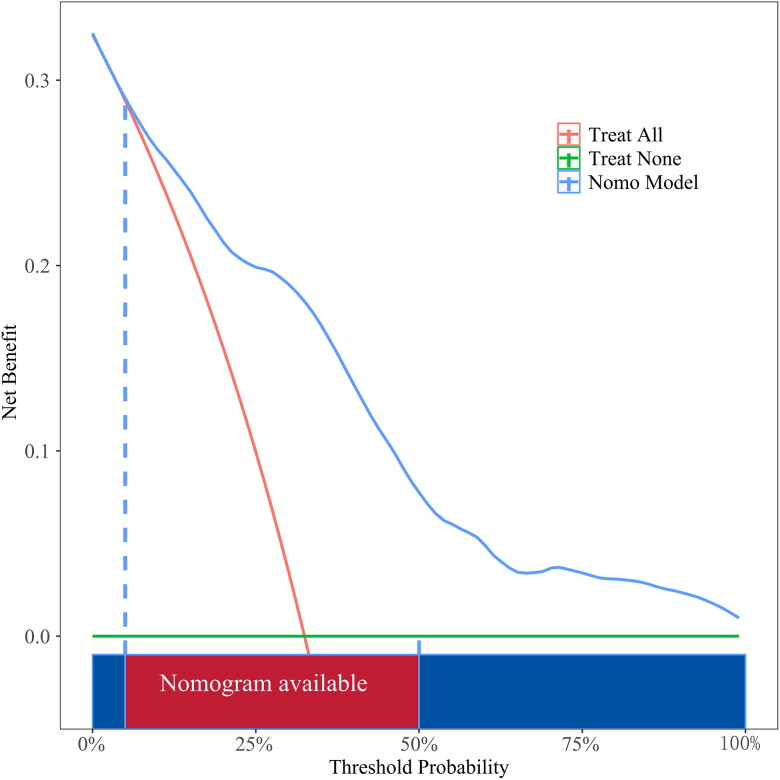
Decision curve analysis plot of the nomogram in the validation set.

To evaluate the efficacy of this model in clinical practice, we validated it *via* DCA and found that compared with providing interventions for all patients or for no patients, the nomogram to predict AVF thrombosis provided greater net benefits for a threshold probability between 5% and 100% (Figures 5 and 6).

## 4. Discussion

For MHD patients, identifying the risk of AVF thrombosis and providing early interventions can prevent thrombosis and reduce patients’ suffering. Therefore, it is necessary to develop a predictive model to identify this risk. Owing to their intuitive visual interface and the ease with which numerical calculations can be performed, nomogram models can help clinicians make better decisions for their patients by avoiding complex equations and operations [26], leading to their widespread use as a prognostic tool in many disciplines.

In this study, a nomogram model was constructed and validated to individually predict patients’ risk of thrombosis in AVFs. The internal verification method was used to verify the model. First, we identified 27 variables to predict the risk of AVF thrombosis in MHD patients. The analysis of these variables showed that the hs-CRP level, intubation history, duration of hemodialysis, AVF stenosis, and non-HDL-C level were risk factors for AVF thrombosis. We then constructed a nomogram prediction model for AVF thrombosis in MHD patients on the basis of the above five predictive variables. The analysis of the model’s performance revealed that its specificity and sensitivity in the training set and the validation set were good and the calibration curves of the nomogram were close to the standard curves, indicating that the model had good calibration ability. DCA further revealed that this model had good net clinical benefits; that is, patients with high levels of hs-CRP, a history of CVC, a long duration of dialysis, AVF stenosis and high levels of non-HDL-C were at greater risk of developing AVF thrombosis. This nomogram model effectively identified patients at risk of developing AVF thrombosis through individualized prediction and allowed the development of early intervention regimens to improve patient outcomes.

Patients with elevated hs-CRP in the blood are considered to be in a state of microinflammation, which changes the structure of the vascular endothelium and subsequently leads to thrombosis [27]. In this study, a high hs-CRP level was an independent predictor of AVF thrombosis. Previous studies employing Kaplan–Meier analysis have found that high levels of hs-CRP could predict AVF thrombosis with a sensitivity of 67.0% and a specificity of 83.7% [28]. Taken together, the findings of the current study and those of the study cited above suggest that the level of hs-CRP can be dynamically monitored in clinical practice to identify the risk of AVF thrombosis and develop early prevention and intervention regimens.

Following deep vein catheterization, friction between the catheter and the vascular wall may damage the vascular endothelial structure, potentially causing vascular stenosis and subsequently leading to thrombosis of the AVF at the corresponding limb [29]. The thrombosis prediction model created in this study revealed that patients with a history of CVC were more likely to experience AVF thrombosis. Therefore, it is important to evaluate the need for catheter use and the possibility of early CVC removal. During the perioperative period, the AVF should be prepared in advance according to the patient’s glomerular filtration rate and the patient’s specific conditions to reduce the use of CVCs and prevent thrombosis [30].

Previous studies [31] have shown that AVFs have 1-, 2-, 3-, 5-, and 10-year patency rates of 77.81%, 73.05%, 64.64%, 60.75%, and 47.48%, respectively, and that these rates are strongly affected by the development of thrombosis. As the time from the formation of the AVF increases, the blood vessel wall gradually becomes more damaged due to repeated punctures. This may eventually lead to intimal hyperplasia [32], which may ultimately increase patients’ risk of thrombosis.

Some studies [33] have suggested that injury to the vessels involved in the AVF is likely to lead to intimal thickening, stimulate the proliferation of collagen fibers in the vascular media, and result in fibroblast accumulation in the outer membrane, causing lumen stenosis and hemodynamic changes leading to thrombosis. Moreover, changes in the shear force in the intima of the vein can lead to thickening of the media, further increasing the degree of lumen stenosis and aggravating the thrombosis. Other studies have shown that almost all cases of internal fistula thrombosis are related to vascular access stenosis [29,34]. The results of this study revealed that vascular stenosis is a risk factor for thrombosis. This could be avoided by better monitoring by nurses of vascular access and by conducting arm lift and pulsation enhancement tests to determine whether the outflow and inflow tracts of the AVF are stenotic [35]. This assessment should also include the use of B-ultrasound examinations to allow the early identification and diagnosis of vascular access stenosis and the early administration of balloon dilation intervention regimens.

Hypercoagulability caused by changes in blood composition can easily result in AVF thrombosis [36]. Studies [6] have shown that AVF thrombosis is closely related to blood lipid status, including the levels of low-density lipoprotein cholesterol (LDL-C) and high-density lipoprotein cholesterol (HDL-C). In this study, we found that non-HDL-C was a risk factor for the development of AVF thrombosis. The American Heart Association has proposed ‘Life’s Essential 8’ [22], including attention to the level of non-HDL-C, which mostly consists of LDL-C [37] and is not strongly affected by diet. In clinical work, nurses should provide timely guidance on medications, diet and exercise to patients with dyslipidemia.

## 5. Strengths and limitations

Strengths of this study are that the sample size was relatively large and that the five variables used to construct the model can be obtained easily in clinical practice and monitored conveniently without additional examination costs. For example, non-HDL-C is not affected by diet; therefore, patients do not need to fast, and the levels can be measured conveniently with blood collection.

However, this study also has several limitations. Some studies have shown that a history of diabetes is a risk factor for thrombosis. It is believed that hyperglycemia can cause damage to the vascular intima, the accumulation of material in the tube wall and the formation of atherosclerotic plaque, which leads to atherosclerosis and thrombosis [38]. In this study, thrombosis had little influence on diabetes history compared with the lack of thrombosis. This finding may be related to the fact that the overall blood sugar level of patients with diabetic nephropathy in our center was well controlled and the overall difference in blood sugar between the two groups was not significant. Some studies have confirmed that hypotension in hemodialysis patients is associated with a greater risk of AVF thrombosis, which may be related to the associated vascular lumen collapse and reduced blood flow [39]. However, the results of this study indicated that hypotension was not an independent risk factor for thrombosis. This finding may be related to a relative lack of data. Our set included patients with hypotension who were undergoing hemodialysis, but many patients experience thrombosis at home without active blood pressure monitoring. Furthermore, this was a single-center retrospective study, and an internal validation set was used to validate the performance of the model. In the future, multicenter prospective studies and external validation are needed to further optimize the model and determine its generalizability to different populations.

## 6. Conclusions

A nomogram was constructed in this study to predict AVF thrombosis in MHD patients. The results showed that hs-CRP, history of CVC, hemodialysis duration, AVF stenosis, and non-HDL-C were risk factors for AVF thrombosis. To identify high-risk patients with AVF thrombosis and provide early intervention to reduce the occurrence of thrombosis, medical staff should increase monitoring of high-risk individuals, and adopt early preventive measures to prevent the formation of AVF thrombi.
